# Genome Sequence of *Salmonella enterica* Phage Det7

**DOI:** 10.1128/genomeA.00279-15

**Published:** 2015-05-07

**Authors:** Sherwood R. Casjens, Deborah Jacobs-Sera, Graham F. Hatfull, Roger W. Hendrix

**Affiliations:** a Department of Biological Sciences, University of Pittsburgh, Pittsburgh, Pennsylvania, USA; b Biology Department, University of Utah, Salt Lake City, Utah, USA; c Pathology Department, Division of Microbiology and Immunology, University of Utah School of Medicine, Salt Lake City, Utah, USA

## Abstract

Det7 is a *Myoviridae* bacteriophage that gains entry into its *Salmonella enterica* serovar Typhimurium host cells by adsorbing to O-antigen polysaccharide. We report here the complete 157,498-bp sequence of its genome. Det7, together with its Vi01-like relatives, are distantly related to phage T4.

## GENOME ANNOUNCEMENT

The virulent double-stranded DNA (dsDNA) bacteriophage Det7 infects *Salmonella enterica* serovar Typhimurium. It has an isometric head that is about 90 nm in diameter and a contractile tail about 120 nm long. Its tail baseplate has numerous brushy protrusions, suggesting the presence of a number of host receptor-binding tailspikes. It adsorbs to *Salmonella* Typhimurium host cells by binding to and cleaving to their surface O-antigen polysaccharide (1).

Det7 was propagated on *S. enterica* LT2 strain DB7000 (2), and its DNA was sequenced using previously described methods (3). In brief, assembly of Sanger sequences of 1,727 randomly chosen clones generated 9 contigs with 31-fold redundancy. Using a total of 245 oligonucleotide primers on Det7 genomic template DNA, these were joined into a single circular contig, and all sequence ambiguities were resolved. The Det7 genome is 157,498 bp long and has 44.6% G+C content. The circular sequence assembly and the similarity of its large terminase subunit (TerL) (32% identical and 48% similar amino acids) to those of phage T4 strongly suggest that Det7 virion DNA is terminally redundant and circularly permuted as the result of a headful packaging mechanism (4).

We identified 210 predicted protein-coding genes in the Det7 genome, 61 of which have a predicted function, as well as five tRNA genes, four of which have products that should recognize the AUG (Met), AAC (Asn), UAC (Tyr), and AGC (Ser) codons. A number of its genes are distant but recognizable homologues of phage T4 core genes (e.g., Det7 major capsid protein amino acid sequence is 29% identical to that of T4, and their DNA polymerases are 26% identical). An analysis of the genome shows that it is a typical highly syntenic member of the Vi01-like phage cluster/genus (5, 6), a group of rather closely related phages that are known to infect several genera of the bacterial family *Enterobacteriaceae*. The circular Det7 genome sequence is opened in the first noncoding sequence upstream of the terminase genes. Det7 is the 21st cluster member whose complete or nearly complete genome sequence has been reported (5, 6). As has been discussed, a notable feature of this phage group is that its members apparently encode several different tailspikes that should allow them to infect multiple host species or serovars. Det7 appears to encode four such proteins. One of these is similar to the phage P22 tailspike that binds *Salmonella* serovar Typhimurium O antigen (1), and the other three are unique in this group of phages, suggesting that Det7 has a different host range than that of the known members of this group. Alternate Det7 hosts may be hinted at by the closest relatives of the C-terminal putative receptor binding domains of the latter three tailspikes that are between 33% and 45% identical to *S. enterica* serovar Anatum phage epsilon15 and prophages in *Escherichia fergusonii* ECD227 (GenBank accession no. CM001142), *Klebsiella* sp. 10982 (accession no. WP_025713874), and *Tolumonas* sp. BRL6-1 (accession no. WP_024873167).

### Nucleotide sequence accession number. 

The complete genome sequence of phage Det7 is available in GenBank with accession no. KP797973.
